# Acceptance of the Administration of Multiple Injectable Vaccines in a Single Immunization Visit in Albania

**DOI:** 10.1093/infdis/jiw570

**Published:** 2017-06-30

**Authors:** Iria Preza, Saleena Subaiya, Jennifer B. Harris, Daniel C. Ehlman, Kathleen Wannemuehler, Aaron S. Wallace, Shahin Huseynov, Terri B. Hyde, Erida Nelaj, Silvia Bino, Lee M. Hampton

**Affiliations:** 1 Institute of Public Health, Tirana, Albania;; 2 Epidemic Intelligence Service, and; 3 Global Immunization Division, Centers for Disease Control and Prevention, Atlanta, Georgia; and; 4 World Health Organization Regional Office for Europe, Copenhagen, Denmark

**Keywords:** multiple injections, inactivated polio vaccine, IPV, Albania, vaccine acceptance, vaccine administration.

## Abstract

**Background.:**

Albania introduced inactivated polio vaccine (IPV) into its immunization system in May 2014, increasing the maximum recommended number of injectable vaccines given in a single visit from 2 to 3.

**Methods.:**

Health-care providers and caregivers were interviewed at 42 health facilities in Albania to assess knowledge, attitudes, and practices regarding injectable vaccine administration. Immunization register data were abstracted from December 2014 to July 2015 at the same facilities to explore the number of injectable vaccines children received during their 2- and 4-month visits.

**Results.:**

The majority of children (87%) identified in the record review at either their 2- or 4-month immunization visit received all 3 injectable vaccines in a single visit. Almost all children who did not receive the vaccines in a single visit were subsequently fully immunized, most within a 2-week period. Over half of caregivers whose children got 3 or more injectable vaccines in a single visit reported being only comfortable with 1 or 2 injectable vaccines in a single visit.

**Conclusions.:**

Despite most caregivers expressing hesitation regarding children receiving multiple injectable vaccines in a single visit, most children received vaccines according to the recommended schedule. Almost all children eventually received all recommended vaccines.

## BACKGROUND

Albania introduced inactivated polio vaccine (IPV) into its routine immunization system in May 2014 as recommended by the World Health Organization’s (WHO’s) Strategic Advisory Group of Experts (SAGE) [1].

This was part of preparation for the April 2016 global switch from trivalent oral polio vaccine (tOPV), which protects against types 1, 2, and 3 polioviruses, to bivalent oral polio vaccine (bOPV), which only protects against types 1 and 3 polioviruses [2, 3]. Like many other countries that introduced IPV in anticipation of the switch from tOPV to bOPV, Albania added doses of standalone IPV to the 2- and 4-month routine immunization visits at which pneumococcal conjugate vaccine (PCV) and pentavalent vaccine (containing diphtheria, tetanus, pertussis, hepatitis B, and *Haemophilus influenzae* type b [Hib] antigens) were also given [4]. As a result, the number of recommended injectable vaccines at those visits increased from 2 to 3.

As the number of injectable vaccines grows, reports from other countries, including European countries, have indicated that some health-care providers and caregivers of children have expressed reservations about children receiving more injections in a single visit [5]. For example, in the Eastern European country of Ukraine, an evaluation of the introduction of Hib vaccine stated that some parents had opposed administration of the vaccine to their children because they did not want their children to receive an additional injection [6]. However, it has also been shown that despite reservations surrounding the administration of multiple injectable vaccines in a single visit, health-care providers and caregivers will often comply with vaccination guidelines [5]. No studies regarding health-care providers’ and children’s caregivers’ acceptance of the administration of multiple injectable vaccines in a single visit have been performed in countries with a public health system similar to that of Albania or other formerly communist Eastern European countries. We conducted a survey of children’s caregivers and public health-care providers in Albania and reviewed immunization register data in order to assess knowledge and attitudes regarding the administration of multiple injectable vaccines at a single visit, and to assess the number of vaccines children received during the visits at which 3 injectable vaccines were recommended.

## METHODS

Stratified simple random sampling was used as the first stage of sampling to select 42 of the 523 health facilities providing immunizations as part of Albania’s National Immunization Program for inclusion in the study. Health facilities were stratified based on location, resulting in 9 selected from urban facilities, 8 selected from rural facilities with daily immunization sessions, and 25 from rural facilities unlikely to have daily immunization sessions. Allocation was based on the proportion of health facilities in each stratum. To better understand when children attending their 2- and 4-month immunization visits actually received IPV, PCV, and pentavalent doses due at those visits, immunization register records for all such children at the selected health facilities were abstracted and reviewed from December 2014 to July 2015. At each facility, health-care providers who had conducted at least 1 routine immunization session in the prior month were interviewed. The sample can be considered self-weighting because both stages were equal-probability sampling. A convenience sample of caregivers of children who had attended their 2- or 4-month immunization visits at the selected facilities were interviewed with the goal of completing 5–11 interviews (depending on facility type) to ascertain their knowledge and attitudes toward vaccination and the vaccines their children received. All interviews were conducted by staff from the South East European Center for Surveillance and Control of Infectious Diseases.

Vaccination coverage and 95% (Wilson) confidence intervals (CIs), accounting for the stratified cluster design, were calculated from the immunization register data. In addition, inverse Kaplan–Meier curves were estimated independently for each of the 3 recommended vaccines for both the 2- and 4-month immunization visits. The curves estimate the time from birth to receipt of each vaccine; children that did not receive a vaccine were censored at the date of their last contact in the immunization register. Among those receiving all 3 antigens but not receiving all 3 in a single visit, the median and interquartile range of days to receiving all recommended vaccines were calculated without accounting for study design. Analyses were conducted using SAS 9.3 (SAS Institute, Inc, Cary, NC) and graphed in R 3.0 (R Foundation for Statistical Computing).

Descriptive results are provided and include demographics of caregivers and health-care providers, vaccination status of children, and attitudes and practices surrounding multiple injections. Immunization data from the record review were used to stratify health facilities into those where >90% of children had received 3 vaccines at their 2- and 4-month visits, and those where ≤90% of children did not.

As this assessment was classified as a routine public health program evaluation by both the Institute of Public Health in Albania and the Centers for Disease Control and Prevention in the United States, a human subject review determination was made but institutional review board approval was not required. Oral informed consent was obtained from participating health-care providers and child caregivers before their interviews. Data were stored on a password-protected device without patient identifiers.

## RESULTS

### Record Reviews

Immunization register records of 1147 children who received their 2- and/or 4-month vaccinations between December 2014 and July 2015 at 42 health facilities were reviewed. Of the 1068 children who were eligible to have received their 2-month vaccinations by the time of the record review, 1064 (99.6%; 95% CI [99%−99.9%]) had received the first dose of IPV (IPV1) and the first dose of PCV (PCV1); all (100%) had received the first dose of pentavalent vaccine (Penta1). Of the 1061 children who had received IPV1, PCV1, and Penta1, 924 (87%; 95% CI [71%–94%]) received all 3 at the same visit, while the remainder received the vaccines over 2 or 3 visits.

**Figure 1.**
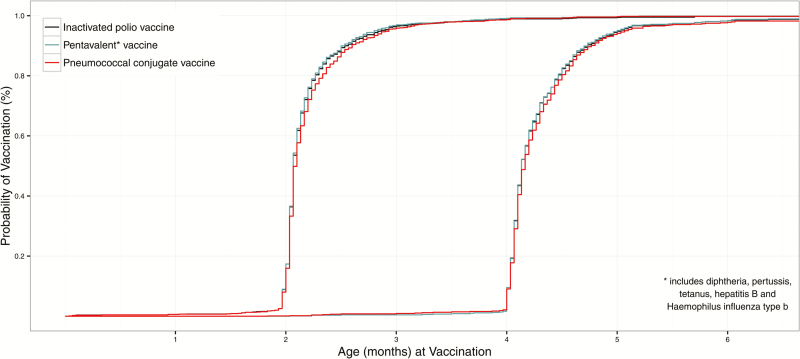
Time from birth to vaccination with first (n=1068) and second (n=726) doses of inactivated polio vaccine, pentavalent vaccine, or pneumococcal conjugate vaccine from retrospective record review.

Most children who did not receive IPV1, PCV1, and Penta1 at the same visit received all 3 vaccines soon after the first visit at which they were eligible for those vaccines (Figure 1). Of the 144 children who did not receive all 3 vaccines at the same visit, 115 received IPV1 and Penta1 but did not receive PCV1 at the first visit when they were eligible for those vaccines, of which 112 (97%) of these children received PCV1 within a median of 6 days (interquartile range [IQR], 3–10 days); the remaining 3 children did not receive PCV1. Another 23 children received PCV1 with either IPV1 or Penta1, of which 19 (83%) received their third vaccination within a median period of 6 days (IQR, 4– 11 days). The remaining 4 children did not receive their third vaccine. The 6 children who received 3 vaccines on 3 separate days were all fully vaccinated within a median period of 46 days (IQR, 15–76 days).

Out of the 726 children who were eligible to have received their 4-month vaccinations by the time of the record review, 723 (99.6%; 95% CI [98%−99.9%]) had been vaccinated with the second dose of IPV (IPV2) and the second dose of pentavalent vaccine (Penta2); 724 (99.7%; 95% CI [99%−99.9%]) had been vaccinated with the second dose of PCV (PCV2). Six hundred thirty-four (87%; 95% CI [75%−94%]) of the eligible children received all 3 at the same visit.

Most of the children who did not receive IPV2, PCV2, and Penta2 in a single visit received all 3 vaccines relatively soon after the first visit at which they were eligible for those vaccines (Figure 1). Of the 92 children who did not receive all 3 vaccines at one time, 71 received IPV2 and Penta2 but not PCV2 at the first visit when they were eligible for those vaccines. Sixty-seven (94%) of these children received PCV2 within a median of 7 days (IQR, 3–15 days); the remaining 4 children did not receive PCV2. Another 14 children received PCV2 with either IPV2 or Penta2, and 13 (93%) received the third vaccine within a median of 7 days (IQR, 6–12 days); the remaining child did not receive the third vaccine. Finally, 6 (86%) of the 7 children that received the 3 vaccines on 3 separate days were fully vaccinated over a median period of 49 days (IQR, 8–61 days); the remaining child did not receive all 3 vaccines during the period of data collection.

### Caregiver and Health-care Provider Interviews

Two hundred eighty-eight caregivers completed interviews, resulting in a median of 5 interviews per facility, with a range of 4–11. Almost all (97%) interviewed caregivers were the child’s mother, over two-thirds (70%) were between 21 and 30 years old, almost half (43%) had an education level of primary school or lower, and approximately one-quarter (27%) had attended university (Table 1). Almost half (46%) of caregivers had only 1 child, 103 (36%) had 2 children, and 51 (18%) had 3 or more children.

**Table 1.** Demographic Characteristics of Interviewed Caregivers of Children and Health-Care Providers

Demographics of Caregivers	N (%) N=288^a^
Age in years	
≤20	17 (6%)
21–25	83 (29%)
26–30	116 (41%)
31–35	41 (14%)
36–40	15 (5%)
≥41	13 (5%)
Education	
No education	2 (1%)
Primary (some or completed)	121 (42%)
Secondary	85 (30%)
University	77 (27%)
Number of children in household	
1	133 (46%)
2	103 (36%)
≥3	51 (18%)
Relationship to child	
Mother	278 (97%)
Father	3 (1%)
Grandparent	7 (2%)
**Demographics of Health-Care Providers**	**N (%**) **N=58**
Position	
Staff nurses	54 (93%)
Auxiliary nurse midwife	4 (7%)
Years worked as vaccinator	
<1 year	9 (16%)
1–5 years	10 (17%)
6–10 years	3 (5%)
>10 years	36 (62%)
**Health Facility Type**	
Urban	10 (17%)
Rural with daily immunization sessions	13 (22%)
Rural with nondaily immunization sessions	35 (60%)

^a^Due to missing data, for age and education, N=285; for number of children in household, N=287.

Two hundred (70%) caregivers said they were only comfortable with 1 or 2 injections per visit, and 33 (12%) reported being comfortable with 3 or more injections per visit (Table 2). Of the 206 caregivers who permitted their child to receive 3 recommended vaccines in a single visit, 131 (64%) reported only being comfortable with 1 or 2 injections, 32 (16%) reported being comfortable with 3 or more, and 43 (21%) reported being comfortable with whatever their child’s health-care provider recommended. Additionally, almost all (98%) caregivers reported that health-care providers are a main source of information to help them decide whether to vaccinate their child and are the main resource they use to determine whether their child should get more than 1 vaccine in a single visit.

**Table 2.** Caregivers’ Comfort Level With Multiple Injections

Maximum Number of Vaccine Injections During 1 Visit That Respondent is Comfortable With Their Child Receiving	All Caregivers (N = 287)^a^	Caregivers Whose Children Received 3 Vaccines at 1 Visit (N=206)	Caregivers Whose Children Did Not Receive 3 Vaccines at 1 Visit (N = 81)
1 or 2 injections	200 (70%)	131 (64%)	69 (85%)
3 or more injections	33 (12%)	32 (16%)	1 (1%)
Whatever my health-care provider recommends	54 (19%)^b^	43 (21%)^b^	11 (14%)

^a^Response not collected for 1 caregiver on this question.

^b^Due to rounding percentages presented, does not add up to 100.

Fifty-eight health-care providers completed interviews (Table 1). Most (93%) of the health-care providers were staff nurses, and the remainder (7%) were midwives. Over half (62%) had worked for more than 10 years as a vaccinator, and the majority (83%) worked in a rural health facility.

Of the 58 interviewed health-care providers, 4 (7%) stated they were only comfortable giving 1 injectable vaccine per session, 32 (55%) stated they were only comfortable giving 2, and 22 (38%) stated they were comfortable with 3 or more (Table 3). Twenty-seven (47%) health-care providers came from 21 facilities at which >90% of the children sampled received 3 injectable vaccines in a single visit, and 31 (53%) came from 21 facilities at which ≤90% of the children sampled received 3 injectable vaccines in a single visit. Of these, there were 7 facilities, all of which were rural and had a relatively small number of immunization visits (median, 3; range, 3–9), where children never received 3 vaccines in a single visit. Only one-quarter (26%) of the health-care providers that came from a health facility where ≤90% of children received 3 injections in a single visit reported being comfortable administering 3 or more injections at once. Over half (52%) of the health-care providers that came from a health facility where >90% of children received 3 injections in a single visit reported being comfortable administering 3 or more injections at once.

**Table 3.** Health-Care Providers’ Comfort Level With Multiple Injections

Maximum Number of Vaccine Injections During 1 Visit That Health-Care Provider Is Comfortable Administering	All Health-Care Providers (N=58)	Health-Care Providers That Came From a Health Facility Where >90% of Children Received 3 Injections at 1 Visit (N=27)	Health-Care Providers That Came From a Health Facility Where ≤90% of Children Received 3 Injections at 1 Visit (N=31)
1 injection	4 (7%)	2 (7%)	2 (6%)
2 injections	32 (55%)	11 (41%)	21 (68%)
3 or more injections	22 (38%)	14 (52%)	8 (26%)

When asked about their perceptions of vaccines, 55 (19%) caregivers and 7 (12%) health-care providers felt that children receive more vaccines than are necessary (Table 4). However, almost all caregivers (98%) as well as all health-care providers (100%) recognized the benefits of immunization. Two-thirds (65%) of caregivers and three-quarters of health-care providers (77%) felt that it would be better for a child to get 3 injectable vaccines in 1 visit if it meant they would be better protected against disease. Finally, only 5 (9%) health-care providers felt that caregivers should be given the liberty to choose which vaccines their child receives.

**Table 4.** Health-Care Provider and Caregivers’ Perceptions Regarding Immunizations and Multiple Injections

Questions	% Caregivers Who Agree N = 288^a^	% Health-Care Providers Who Agree N = 58^a^
Children get more vaccinations than are necessary^b^	55 (19%)	7 (12%)
Immunizations do more good than harm	279 (98%)	58 (100%)
Many of the illnesses which vaccinations prevent are severe	282 (99%)	56 (97%)
It is better for a child to receive more injectable vaccines at a single visit if it means that they will be better protected against diseases	186 (65%)	44 (77%)
There will be fewer side effects if a child receives 1 injectable vaccine in multiple separate visits rather than multiple injections in a single visit	129 (45%)	16 (28%)
Parents should be allowed to selectively choose the vaccines which they believe their children needs	…^c^	5 (9%)

^a^Percentages may vary due to number of respondents who answered each question.

^b^“Strongly agree” and “agree” were combined. Percentages may vary due to number of respondents who answered each question.

^c^This question was not asked of caregivers.

## DISCUSSION

The majority of health-care providers and caregivers reported being more comfortable with children receiving only 1 or 2 injectable vaccines per visit, yet most (>85%) children whose records were reviewed from both the 2- and 4-month visits received all 3 recommended injectable vaccines in a single visit. Additionally, almost all children who did not receive the recommended 3 injectable vaccines in a single visit subsequently received the remaining recommended vaccines. This suggests that despite stated health-care provider and caregiver preferences regarding multiple injections, most health-care providers and caregivers are willing to follow vaccination guidelines. Furthermore, despite some deviations from the recommended dosing schedules, vaccination coverage was high.

The clustering by health facility of children who did and did not receive all 3 injections at 1 visit suggests that health-care providers play a key role in determining how many injectable vaccines children receive. At many health facilities, over 90% of the children whose records were reviewed received 3 injectable vaccines at the appropriate visits. At these facilities, over half (52%) of health-care providers felt comfortable giving 3 or more injections at 1 visit. In contrast, at the facilities where <90% of children received 3 or more injections at 1 visit almost three-quarters of health-care providers reported being only comfortable administering 1 or 2 injections. Health-care providers also demonstrated willingness to exercise authority with regard to vaccination, with only 9% stating they believed caregivers should be able to select which vaccines their children receive. Our findings support the results of other studies conducted in the European region, demonstrating that caregivers appear to trust health-care providers, with the majority reporting that they were a main source of information regarding how their children should be vaccinated [7–9]. Consistent with the findings of previous studies in countries in other regions of the world [3, 5], the providers’ influence underscores the importance of carefully training health-care providers and enlisting their help in parent outreach when introducing a new vaccine or substantially changing immunization policies, as well as the importance of addressing concerns health-care providers may have regarding multiple injections.

Despite our study demonstrating overall high coverage, the immunization records review demonstrated that 1 in 6 children were not vaccinated according to national and WHO recommendations, as 13% of children received the vaccines spread out over 2 or 3 visits or did not receive all 3 recommended vaccines. Fortunately, almost all of the children who did not receive 3 vaccines in a single visit were tracked by the health-care system and received the recommended vaccines within a few weeks. However, Albania’s success in ensuring that children complete vaccinations in a timely manner is likely not generalizable to many other countries, as Albania has very high reported national vaccination coverages: 99% for third dose of vaccine containing diphtheria, tetanus, and pertussis antigens and 98% for first dose of vaccine containing measles antigen in 2014 [10]. This high coverage is the result of a well-organized vaccination program with 523 vaccination centers for a population of roughly 2.9 million people with approximately 40000 births a year [11]. A strong health infrastructure allows for children under 1 year of age to receive 6 home visits per year to check on basic health indicators and ensure that children receive all needed care, including vaccinations. Furthermore, there is strong support for vaccinations and a belief that vaccines protect against serious diseases, which can help reduce the risk of incomplete vaccination for a child when vaccinations are delayed to a subsequent visit. In contrast, in countries where vaccination sessions are infrequent, caregivers have to travel long distances with their children to reach vaccination sites, or stock-outs are common, delayed vaccination could result in incomplete vaccination for a child and lower vaccination coverage on a population level [12].

This study has several limitations. The children’s caregivers interviewed for the study were a convenience sample of children’s caregivers attending one of the 42 health facilities on the day it was visited, so their knowledge, attitudes, and practices related to immunizations, including the administration of multiple injectable vaccines at a single visit, may not be representative of all children’s caregivers in Albania. Interviewing caregivers after their child received multiple injectable vaccines in a single visit in a health facility compared to in their home may have introduced bias. In addition, we do not have documentation of specific factors directly affecting nonreceipt of all 3 vaccines at 1 visit. This may have been affected by issues unrelated to attitudes and practices of caregivers and health-care providers that we were unable to describe.

## CONCLUSIONS

Although 1 in 6 children did not receive doses of IPV, PCV, and pentavalent vaccine together at their 2- or 4-month immunization visits as recommended in Albania, almost all children received all 3 injectable vaccines within 1 or 2 weeks of the visit at which they were first eligible to receive those vaccines. Health-care providers likely played a key role in determining how many vaccines a child received at a single visit, given the trust that children’s caregivers had in health-care providers regarding vaccinations, the belief of almost all health-care providers that caregivers should not decide which vaccines their children should receive, and the clustering of children who did not receive all 3 injectable vaccines in 1 visit at certain facilities. This suggests that health-care provider recommendation may have strong influence in overcoming hesitancy for vaccination, and that providers’ education may be key in ensuring success in the delivery of multiple injectable vaccines in a single visit. Additionally, the Albanian experience illustrates that most children received all 3 recommended injections despite health-care providers’ and child caregivers’ concerns about multiple injectable vaccines at a single visit, which indicates that such concerns need not be a significant barrier to the introduction of new injectable vaccines.

## Notes

***Financial support.*** This work was supported by funds from the U.S. Centers for Disease Control and Prevention.

***Supplement sponsorship.*** This work is part of a supplement coordinated by the Task Force for Global Health with funding provided by The Bill and Melinda Gates Foundation and the Centers for Disease Control and Prevention.

***Potential conflicts of interest.*** All authors: No reported conflicts.
